# Use of non-bound proteinogenic amino acids to modulate the growth of pathogenic bacteria from broiler chickens

**DOI:** 10.1016/j.psj.2025.106121

**Published:** 2025-11-15

**Authors:** David Chau, Conny Turni, Eugeni Roura, Lida Omaleki

**Affiliations:** Queensland Alliance for Agriculture and Food Innovation, The University of Queensland, St Lucia, Queensland 4067, Australia

**Keywords:** Proteinogenic amino acid, pathogen, bacteria, l-cysteine, chicken

## Abstract

Pathogenic bacteria can inhabit the chicken intestinal tract and potentially cause disease and mortality in chickens. Proteinogenic amino acids are major nutrients available in the intestine that have diverse growth-modulating effects on bacteria. However, limited knowledge exists about the direct association between amino acids and the growth of bacteria in the microbiome of the chicken’s intestinal tract.

The present study evaluated the growth-modulating effects of the 20 proteinogenic amino acids on chicken pathogens. Seven bacterial species were tested, including avian pathogenic *Escherichia coli* (APEC), *Salmonella enterica*., *Staphylococcus aureus, Clostridium perfringens, Enterococcus cecorum, Enterococcus faecalis*, and *Enterococcus faecium*. All bacteria were tested in minimal nutrient medium (MNM) in an *in vitro* model, with or without the addition of each amino acid. Bacterial growth was measured by absorbance (OD_600_) and visualised as growth curves.

l-cysteine significantly (*p* ≤ 0.05) inhibited the growth of APEC, extended the lag phase and increased the maximum density of *C. perfringens, Salmonella enterica*. and *S. aureus*. In contrast, l-glutamine and l-glutamic acid significantly shortened the lag phase and increased both the growth rate and maximum density of *C. perfringens*, Glycine and l-serine significantly (*p* ≤ 0.05) increased the maximum density of *S. aureus*. The effect of amino acids was insignificant (*p* ≥ 0.05) on the total growth of *Enterococcus* spp.

These findings suggested that l-cysteine has inhibiting effects on APEC, *C. perfringens, S. enterica* and *S. aureus*. l-glutamine and l-glutamic acid promoted the growth of *C. perfringens*, while glycine and l-serine promoted the growth of *S. aureus*.

## Highlight

• L-cysteine suppresses the growth of APEC, *C. perfringens, S. enterica*, and *S. aureus* at 1 g/L.
• L-glutamine and l-glutamic acid promote the growth of *C. perfringens.*
• Glycine and L‑serine promote the growth of *S. aureus.*
• The 20 proteinogenic amino acids have no significant effect on the growth of *Enterococcus cecorum, E. faecalis* and *E. faecium.*

## Introduction

Chickens can be inhabited by over a thousand bacterial species in the intestinal tract. This large bacterial population can include pathogenic bacteria (Devriese et al., 1991; Syed et al., 2020; Foster et al., 2021). Several species, *Escherichia coli, Salmonella enterica*., *Staphylococcus aureus* (Swayne, 2020), *Clostridium perfringens* (Foster et al., 2021) and *Enterococcus* species *(E. cecorum, E. faecalis* and *E. faecium*) (Souillard et al., 2022), can be associated with intestinal or extraintestinal diseases. These diseases can be associated with the overgrowth and translocation of bacteria (Milgroom, 2023). The bacteria can lead to disease symptoms such as diarrhoea, lethargy and death (Swayne, 2020). To control these risks, various feeding strategies have been studied to raise chickens to be more disease-resistant, including using feed additives, micronutrients, enzymes and amino acids (Adedokun and Olojede, 2019). While these strategies target the chicken itself, a strategy has been proposed that feeds the intestinal bacteria directly and modulates the growth of a specific group of bacteria (Beaumont et al., 2022).

The availability of some essential nutrients can promote bacterial growth. Like other organisms on Earth, amino acids are the building blocks of the proteins essential for cellular function. However, some bacteria may have limited mechanisms to synthesise some specific amino acids from alternative nitrogen sources. The absence of the amino acids limits bacterial development. An example of the promoting effect of amino acids is the increased abundance of *C. perfringens* in chickens with a fishmeal diet, where the author associated the increased growth of *C. perfringens* with glycine and methionine (Drew et al., 2004). In other words, glycine and methionine can be the limiting amino acids for the growth of *C. perfringens.*

In contrast, amino acids can inhibit bacterial growth. The inhibitory effects of free amino acids were first discussed during the development of bacterial culture medium (Wyon and McLeod, 1923). The authors suggested that protein disintegration during polypeptide preparation leads to the formation of free amino acids. The free amino acids, including histidine, tyrosine, tryptophan and cystine, were found to exhibit inhibitory properties on *S. aureus* at concentrations below 40 mM (Wyon and McLeod, 1923). The inhibitory properties of free amino acids on bacteria have also been discussed for l-cysteine (Kari et al., 1971), l-arginine (Ben-Porat et al., 2024), l-glutamine (Wang et al., 2016), l-isoleucine (Amos and Cohen, 1954), l-serine (Duan et al., 2016) and l-valine (Rowley, 1953; De Felice et al., 1977).

Despite the studies demonstrating the growth-modulating effect of amino acids on bacteria, the specific effects of amino acids on chickens’ intestinal bacteria are a knowledge gap. In commercial broiler feeds, crystalline (also referred to as synthetic) amino acids are commonly supplemented to meet the nutritional requirements for chickens. In experimental settings, the supplementation can range from 0.68 g/kg for tryptophan to 10.10 g/kg for l-lysine in a low crude protein diet (Macelline et al., 2023). Such supplementation not only provides nutrients to the chickens but may also influence the bacterial growth. However, a comprehensive understanding of the direct growth-modulating effect of individual amino acids on chickens’ bacteria is still lacking.

Minimal nutrient medium (MNM) has been widely used to study microbial physiological responses and nutritional requirements (Alazzam et al., 2011; Machado et al., 2019; Kwoji et al., 2022). The advantage of using MNM is that confounding factors can be minimised. Many studies have made conclusions about the bacterial growth-modulating effect from *in vivo* experiments (Dahiya et al., 2007; U. Bello et al., 2018), where confounding factors, including communication within the microbiota (Mukherjee and Bassler, 2019), the diet of the host, the immune status of the host (Kers et al., 2018) and the housing environment (Hubert et al., 2019), can interfere with the result. These influences are minimal in MNM. As a result, MNM provide a valuable tool to evaluate the direct effects of amino acids on bacterial growth. In this study, we hypothesised that, among the 20 proteinogenic amino acids, some of them inhibit and some of them promote the growth of chickens’ bacterial pathogens, including Avian Pathogenic *E. coli* (APEC), *C. perfringens, S enterica, S. aureus, E. cecorum, E. faecalis* and *E. faecium*, in the MNM.

## Materials and Methods

### Bacteria isolates and culture conditions

This study included isolates from seven bacterial species obtained from clinical cases of broiler chickens. Details regarding the origin of each isolate are provided in Supplementary Table 1, where available. This collection comprises eight APEC isolates, as described in Thomrongsuwannakij et al. (2020), as well as five *C. perfringens*, five *S. enterica,* six *S. aureus*, four *E. cecorum*, five *E. faecalis* and five *E. faecium* isolates. The five *S. enterica* isolates represented different serovars: S39 (Bredeney), S41 (Hessarek), S44 (Typhimurium), S116 (Adelaide) and S175 (Saintpaul). All isolates were stored in Protect (Technical Service Consultants Ltd, UK) at –80 °C. The bacteria were revived on sheep blood agar and grew at 37 °C for 24 hours in aerobic conditions except for *C. perfringens*, which was in a sealed container with AnaeroGen 3.5 L Sachet (Oxoid, Basingstoke, UK).

### Amino acid auxanography

The growth dynamics of each bacterial species were tested in 96-well plates (Thermo Fisher Scientific, USA). To evaluate all potential physiological effects of proteinogenic amino acids, the bacteria were cultured in their respective minimum nutrient media: M9 medium for *E. coli* and *S. enterica* (Sambrook and Russell, 2001), a modified M9 medium for *Enterococcus* species (Geldart and Kaznessis, 2017) and a synthetic minimum nutrient medium for *S. aureus* (Rudin et al., 1974). *C. perfringens* was cultured in a medium described by Fuchs and Bonde (1957), supplemented with 37.8 mg/L glycine, 109 mg/L l-lysine, 83 mg/L l-methionine and 53.2 mg/L l-serine (Fuchs and Bonde, 1957), as well as 1 mg/L nicotinic acid, 0.5 mg/L riboflavin, 1 mg/L thiamine and 10 mg/L uracil (Riha and Solberg, 1971). For simplicity, all these distinct minimal media are collectively referred to as MNM throughout this paper.

The twenty proteinogenic amino acids (L-alanine (Ala), l-arginine (Arg), l-asparagine (Asn), l-aspartate (Asp), l-cysteine (Cys), l-glutamine (Gln), l-glutamate (Glu), glycine (Gly), l-histidine (His), l-isoleucine (Ile), l-leucine (Leu), l-lysine (Lys), l-methionine (Met), l-phenylalanine (Phy), l-proline (Pro), L‑serine (Ser), l-threonine (Thr), l-tryptophan (Trp), l-tyrosine (Tyr) and l-valine (Val)) (Sigma-Aldrich, USA) were tested at a concentration of 1 g/L when added to MNM. For nineteen of the amino acids, 2 g/L stock solutions were prepared by dissolving them in water. However, in the case of Tyr, a 10 g/L stock solution was prepared using 0.2 N NaOH as solvent. All the amino acid solutions were filter sterilised using 0.2 µm syringe SCFA filters (Thermo Fisher Scientific, USA). Bacterial isolates from the overnight culture on sheep blood agar were suspended directly in the MNM and adjusted to an OD600 of 0.5 with Eppendorf BioPhotometer 6131 (Eppendorf AG, Hamburg, Germany). Then, the suspension was diluted 1 in 50 to prepare the initial inoculum. For *S. aureus*, a 1 in 4 dilution was used instead, as *S. aureus* did not grow when the suspension was diluted 1 in 50. For *C. perfringens*, 0.5 g/L sodium hydrosulphite (Chem-Supply, Australia) was added as a reducing agent and a layer of 120 µl of paraffin oil heavy 68 (Chem-Supply, Australia) was overlaid on each well to keep the medium anaerobic.

All experiments were conducted with three biological replicates. For each replicate, bacterial isolates were grown under 22 different conditions: 20 experimental conditions, each supplemented with a different amino acid, and two control conditions: MNM (without additional amino acids) and MNM+OH (MNM with 0.02 N NaOH, serving as the positive control for Tyr). To account for the background absorbance, 22 corresponding blank wells were prepared, consisting of MNM with each condition but without bacteria. These blanks enabled accurate subtraction of baseline absorbance from the experimental wells. The assays were incubated at 37 °C on the Ratek platform mixer (Ratek Instruments Pty Ltd, Melbourne, Australia) at 200 rpm, except for *C. perfringens,* which was incubated in the FLUOstar Optima microplate reader for 24 hours without shaking to maintain an oxygen-free condition.

### Growth curves

The bacterial growth was estimated by the turbidity of the liquid medium (Koch, 1970). The optical density was measured at 600 nm (OD_600_) (Myers et al., 2013). The OD_600_ readings were recorded every two hours by taking the plates to the FLUOstar Optima microplate reader from 0 to 14 hours, and then at the 24th and 48th hour of incubation. For *C. perfringens*, absorbance was measured every hour for 24 hours. For *S. aureus*, measurements were taken every hour from 7 to 24 hours, and then at the 36th and 48th hour of incubation, as no growth was observed during the first six hours in the preliminary tests. For each condition, datapoints were the final yield, represented by (OD_600_ of well with bacteria) – (OD_600_ of corresponding blank).

### Statistics analysis

Five growth parameters were calculated using the R package “gcplyr” (Blazanin, 2023): the area under the growth curve (AUC)(total growth), initial growth time (the duration in hours to reach OD_600_ of 0.1), lag time (the duration to reach the maximum growth rate), maximum growth rate and maximum density. Statistical analyses were performed using R v4.3.2 (R Core Team, 2023). Methods of statistical comparisons were chosen depending on the normality of the data, which was confirmed by the Shapiro test (Royston, 1982). The Student’s T-test was used to compare normally distributed data, while the Mann-Whitney test was used to compare non-normally distributed data. The *p*-values were then adjusted with the FalseDiscoveryRate (FDR) method (Benjamini and Hochberg, 1995). The changes in the mean (%) of the growth parameters were calculated as $change\;in\;mean\;\left(\%\right)=(\frac{x-y\;}{y}\times \;100$), where x and y are the mean values of the growth parameters, calculated from conditions with and without amino acids, respectively. The results are presented as heatmaps.

## Result

### Avian pathogenic *E. coli*

**Fig 1:**
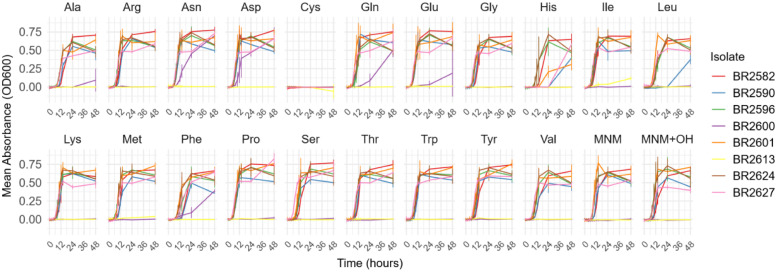
Growth curves of the mean absorbance (OD_600_) of a triplicate against the incubation duration (hour) for 8 APEC isolates in M9 minimal medium with or without 1 g/L of each amino acid. Ala: l-alanine, Arg: l-arginine, Asn: l-asparagine, Asp: l-aspartic acid, Cys: l-cysteine, Gln: l-glutamine, Glu: l-glutamic acid, Gly: glycine, His: l-histidine, Ile: l-isoleucine, Leu: l-leucine, Lys: l-lysine, Met: l-methionine, Phe: l-phenylalanine, Pro: l-proline, Ser: l-serine, Thr: l-threonine, Trp: l-tryptophan, Tyr: l-tyrosine, Val: l-valine, MNM (minimal nutrient medium), MNM+OH (minimal nutrient medium with 0.02*N* NaOH).

All isolates failed to grow in MNM supplemented with Cys (Figure 1). Histidine delayed the growth of BR2590 and BR2627. The growth of BR2590 was also delayed by leucine. Two isolates, BR2600 and BR2613, showed no growth in the unsupplemented MNM medium, hence they were excluded from the statistical analysis. BR2613 grew slightly in the MNM when isoleucine was added, while BR2600 showed growth when Asn, Asp, Gln, Glu or Phe were added. Diauxic shift was observed in the growth curves of BR2627 with asparagine, aspartic acid and proline

**Fig 2:**
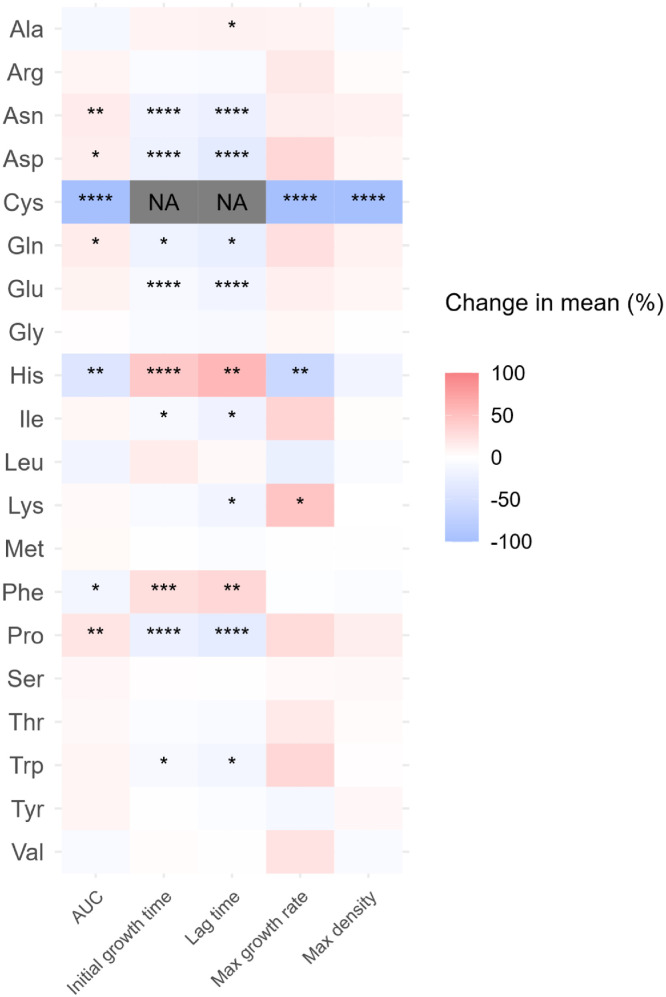
Heatmap presenting the change in mean value of five growth curves parameters (area under the curves (AUC), initial growth time, lag time, maximum growth rate and maximum density) of avian pathogenic *E. coli* (APEC). Ala: l-alanine, Arg: l-arginine, Asn: l-asparagine, Asp: l-aspartic acid, Cys: l-cysteine, Gln: l-glutamine, Glu: l-glutamic acid, Gly: glycine, His: l-histidine, Ile: l-isoleucine, Leu: l-leucine, Lys: l-lysine, Met: l-methionine, Phe: l-Phenylalanine, Pro: l-proline, Ser: l-serine, Thr: l-threonine, Trp: l-tryptophan, Tyr: l-tyrosine, Val: l-valine. *: *p* ≤ 0.05; **: *p* ≤ 0.01; ***: *p* ≤ 0.001; ****: *p* ≤ 0.0001.

The AUC (Figure. 2) increased significantly (*p* ≤ 0.05) with the addition of Asn (15.8 % [3.39 OD₆₀₀·h]), Asp (13.9 % [2.98 OD₆₀₀·h]), Gln (15.4 % [3.30 OD₆₀₀·h]), and Pro (19.9 % [4.25 OD₆₀₀·h]). Asn, Asp, Gln, Glu, Ile, Pro and Trp shortened the initial growth time and lag time by 17.3 % (1.06 hours) and 23.1 % (2.10 hours), 20.0 % (1.22 hours) and 30.7 % (2.78 hours), 19.1 % (1.17 hours) and 24.6 % (2.23 hours), 10.0 % (0.611 hours) and 15.8 % (1.44 hours), 10.0 % (0.611 hours) and 18.3 % (1.66 hours), and 10.0 % (0.611 hours) and 14.6 % (1.33 hours), respectively. Lys decreased the lag time and increased the growth rate by 15.3 % (1.39 hours) and 46.7 % (0.400), respectively.

The AUC was decreased (*p* ≤ 0.05) by Cys (99.87 % [21.4 OD₆₀₀·h]), His (39.9 % [8.52 OD₆₀₀·h]) and Phe (14.8 % [3.1752 OD₆₀₀·h]). Ala increased the lag time by 10.4 % (0.945 hours). Cys completely inhibited the growth of *E. coli*; consequently, the initial growth time and lag time were not measurable, with the maximum growth rate and density recorded as 0.00. His increased the initial growth time and lag time by 43.6 % (2.67 hours) and 57.0 % (5.18 hours), respectively, and decreased the maximum growth rate by 61.7 % (0.528 hour^-1^). Phe increased the initial growth time by 25.5 % (1.56 hours) and lag time by 32.3 % (2.93 hours).

### C. perfringens

**Fig 3:**
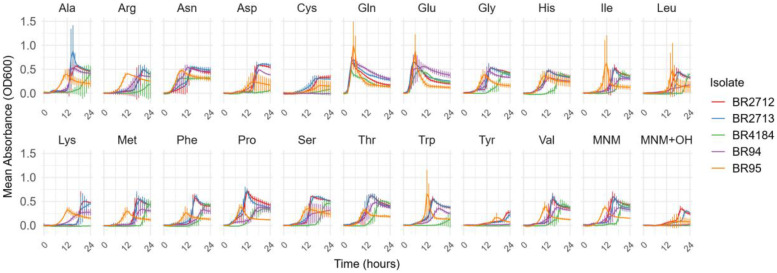
Growth curves of the mean absorbance (OD_600_) of a triplicate against the incubation duration (hour) for 5 *C. perfringens* isolates in the MNM with or without 1 g/L of each amino acid. Ala: l-alanine, Arg: l-arginine, Asn: l-asparagine, Asp: l-aspartic acid, Cys: l-cysteine, Gln: l-glutamine, Glu: l-glutamic acid, Gly: glycine, His: l-histidine, Ile: l-isoleucine, Leu: l-leucine, Lys: l-lysine, Met: l-methionine, Phe: l-phenylalanine, Pro: l-proline, Ser: l-serine, Thr: l-threonine, Trp: l-tryptophan, Tyr: l-tyrosine, Val: l-valine, MNM (minimal nutrient medium), MNM+OH (minimal nutrient medium with 0.02*N* NaOH).

**Fig 4:**
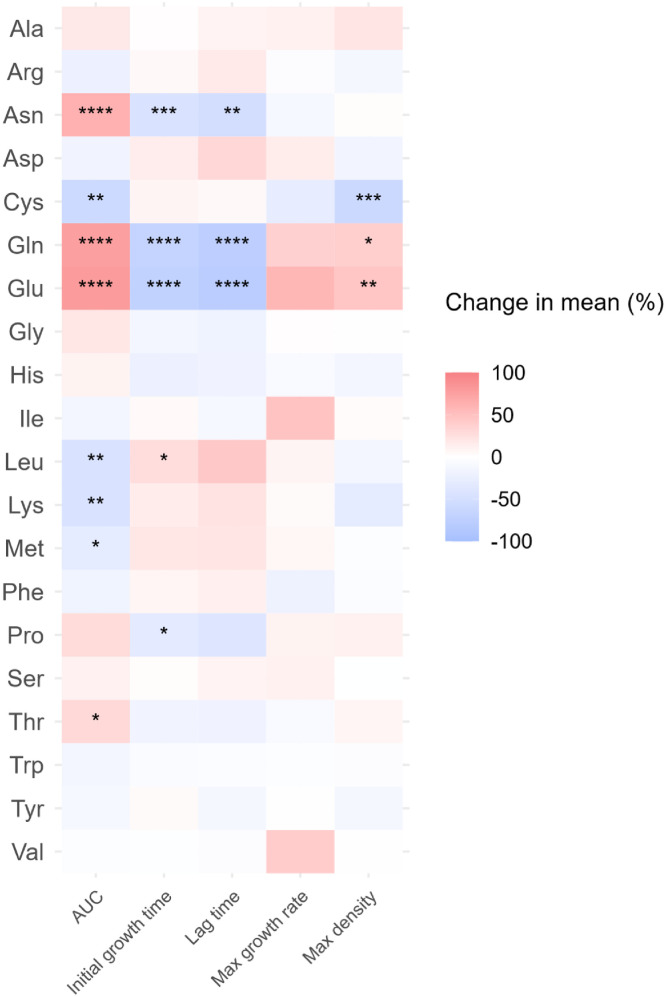
Heatmap presenting the change in mean value of five growth curves parameters (area under the curves (AUC), initial growth time, lag time, maximum growth rate and maximum density) of *C. perfringens*. Ala: l-alanine, Arg: l-arginine, Asn: l-asparagine, Asp: l-aspartic acid, Cys: l-cysteine, Gln: l-glutamine, Glu: l-glutamic acid, Gly: glycine, His: l-histidine, Ile: l-isoleucine, Leu: l-leucine, Lys: l-lysine, Met: l-methionine, Phe: l-phenylalanine, Pro: l-proline, Ser: l-serine, Thr: l-threonine, Trp: l-tryptophan, Tyr: l-tyrosine, Val: l-valine. *: *p* ≤ 0.05; **: *p* ≤ 0.01; ***: *p* ≤ 0.001; ****: *p* ≤ 0.0001.

The five tested *C. perfringens* strains were all able to grow in the MNM. While in the MNM, the growth curves reach a peak at approximately 12 hours of incubation, the growth curves reach the peak at five to six hours of incubation in the presence of Gln or Glu (Figure 3). Also, l-cysteine reduced the peak OD_600_ of all isolates to below 0.5 as demonstrated in Figure 3. The AUC (Figure 4) was significantly increased (*p* ≤ 0.05) with the addition of Asn (61.9 % [2.6352 OD₆₀₀·h]), Gln (79.4 % [3.35 OD₆₀₀·h]), Glu (76.4 % [3.25 OD₆₀₀·h]) and Thr (30.1 % [1.28 OD₆₀₀·h]). Asn reduced the initial growth time by 14.3 % (6.33 hours) and the lag time by 31.7 % (5.22 hours). Gln and Glu decreased the initial growth time by 71.4 % (10 hours) and 67.1 % (9.4 hours), respectively, and the lag time by 79.2 % (8.08 hours) and 74.8 % (7.63 hours), respectively. Additionally, these compounds increased the maximum density by 45.4 % (OD_600_ of 0.218) and 38.2 % (OD_600_ of 0.184), respectively. Pro reduced the initial growth time by 31.9 % (4.47 hours).

Conversely, AUC was reduced by Cys (57.9 % [2.4652 OD₆₀₀·h]), Leu (44.4 % [1.89 OD₆₀₀·h]), Lys (45.0 % [1.92 OD₆₀₀·h]) and Met (29.2 % [1.25 OD₆₀₀·h]). Cys reduced the maximum density by 59.9 % (OD_600_ of 0.288), while Leu shortened the initial growth time by 27.6 % (3.86 hours)

### Enterococcus spp

By visual examination of the growth curves, the overall growth pattern did not have an obvious change in the presence of any of the amino acids (Supplementary Figure 1, Supplementary Figure 3 and Supplementary Figure 5). The variation of growth pattern in the presence of different amino acids could only be observed in some strains, including *E. cecorum* strain BR4158, *E. faecalis* strain BR4144 and *E. faecium* strain BR4148

The AUC for *E. cecorum* decreased with the addition of Tyr by 24.2 % (4.55 OD₆₀₀·h) (Supplementary Figure 2), whereas the AUC for *E. faecium* and *E. faecalis* were not significantly affected (*p* > 0.05) by the addition of any of the amino acids (Supplementary Figure 4 and Supplementary Figure 6).

Cys and Tyr exhibited statistically significant effects (*p* ≤ 0.05) on *E. faecium* and *E. cecorum*, respectively. Cys increased the lag time of *E. faecium* by 4.65 % (0.246 hours) (Supplementary Figure 6). Tyr decreased the maximum density of *E. cecorum* by 25.6 % (OD_600_ of 0.166) (Supplementary Figure 4) and increased the initial growth time of *E. faecium* by 55.9 % (3.8 hours) (Supplementary Figure 6)

### Salmonella enterica

**Fig 5:**
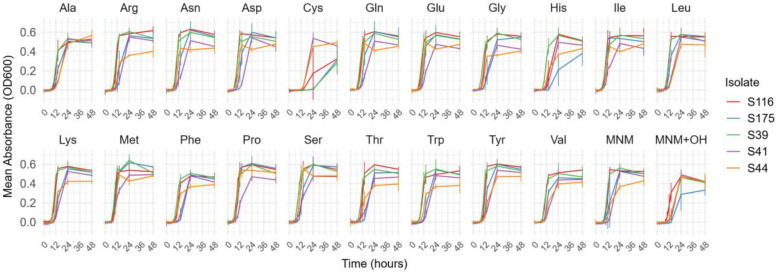
Growth curves of the mean absorbance (OD_600_) of a triplicate against the incubation duration (hour) for 5 *Salmonella* spp. isolates in MNM with or without 1 g/L of each amino acid. Ala: l-alanine, Arg: l-arginine, Asn: l-asparagine, Asp: l-aspartic acid, Cys: l-cysteine, Gln: l-glutamine, Glu: l-glutamic acid, Gly: glycine, His: l-histidine, Ile: l-isoleucine, Leu: l-leucine, Lys: l-lysine, Met: l-methionine, Phe: l-phenylalanine, Pro: l-proline, Ser: l-serine, Thr: l-threonine, Trp: l-tryptophan, Tyr: l-tyrosine, Val: l-valine, MNM (minimal nutrient medium), MNM+OH (minimal nutrient medium with 0.02*N* NaOH).

All five *S. enterica* isolates were able to grow in the MNM. Cys delayed the growth of all isolates except for S41 and S44 as observed in Figure 5.

The AUC increased with the addition of Met (18.4 % [3.0552 OD₆₀₀·h]), Pro (22.2 % [3.6852 OD₆₀₀·h]), Ser (16.8 % [2.7952 OD₆₀₀·h]) and Tyr (43.4 % [5.6152 OD₆₀₀·h]). Ile, Met, Pro and Ser reduced the initial growth time and lag time by 19.6 % (1.47 hours) and 30.7 % (3.76 hours), 21.4 % (1.6 hours) and 30.7 % (3.76 hours), 23.2 % (1.73 hours) and 34.7 % (4.25 hours), and 17.0 % (1.27 hours) and 26.3 % (3.23 hours), respectively.

Tyr reduced the initial growth time by 15.0 % (1.27 hours) and the lag time by 27.3 % (4.01 hours), while increasing the maximum growth rate by 98.5 % (0.524 hour^-1^) and the maximum density by 24.1 % (OD_600_ of 0.109). Additionally, Asn, Gln, Glu and Ile reduced the lag time by 20.9 % (2.56 hours), 24.1 % (2.95 hours), 25.2 % (3.09 hours) and 30.7 % (4.23 hours), respectively.

**Fig 6:**
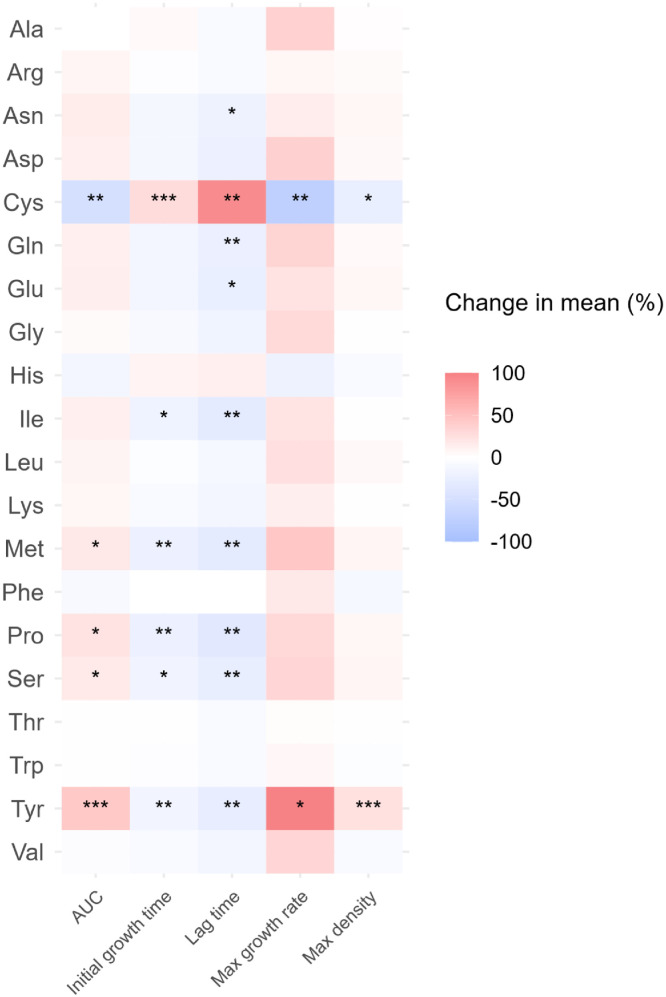
Heatmap presenting the change in mean value of five growth curves parameters (area under the curves (AUC)), initial growth time, lag time, maximum growth rate and maximum density) of *Salmonella* spp.. Ala: l-alanine, Arg: l-arginine, Asn: l-asparagine, Asp: l-aspartic acid, Cys: l-cysteine, Gln: l-glutamine, Glu: l-glutamic acid, Gly: glycine, His: l-histidine, Ile: l-isoleucine, Leu: l-leucine, Lys: l-lysine, Met: l-methionine, Phe: l-Phenylalanine, Pro: l-proline, Ser: l-serine, Thr: l-threonine, Trp: l-tryptophan, Tyr: l-tyrosine, Val: l-valine. *: *p* ≤ 0.05; **: *p* ≤ 0.01; ***: *p* ≤ 0.001; ****: *p* ≤ 0.0001.

Conversely, the AUC decreased with the addition of Cys (49.1 % [8.15 OD₆₀₀·h]). Cys increased the initial growth time by 27.7 % (2.07 hours) and the lag time by 91.8 % (13.3 hours), while reducing the maximum growth rate by 73.6 % (0.599 hour^-1^) and the maximum density by 25.2 % (0.133 OD_600_) as illustrated in Figure 6.

### Staphylococcus aureus

**Fig 7:**
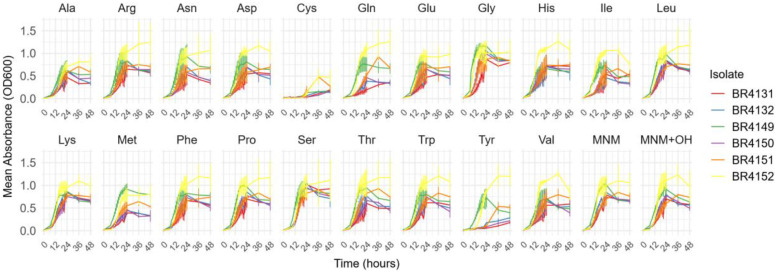
Growth curves of the mean absorbance (OD_600_) of a triplicate against the incubation duration (hour) for 5 *S. aureus* isolates in MNM with or without 1 g/L of each amino acid. Ala: l-alanine, Arg: l-arginine, Asn: l-asparagine, Asp: l-aspartic acid, Cys: l-cysteine, Gln: l-glutamine, Glu: l-glutamic acid, Gly: glycine, His: l-histidine, Ile: l-isoleucine, Leu: l-leucine, Lys: l-lysine, Met: l-methionine, Phe: l-phenylalanine, Pro: l-proline, Ser: l-serine, Thr: l-threonine, Trp: l-tryptophan, Tyr: l-tyrosine, Val: l-valine, MNM (minimal nutrient medium), MNM+OH (minimal nutrient medium with 0.02*N* NaOH).

**Fig 8:**
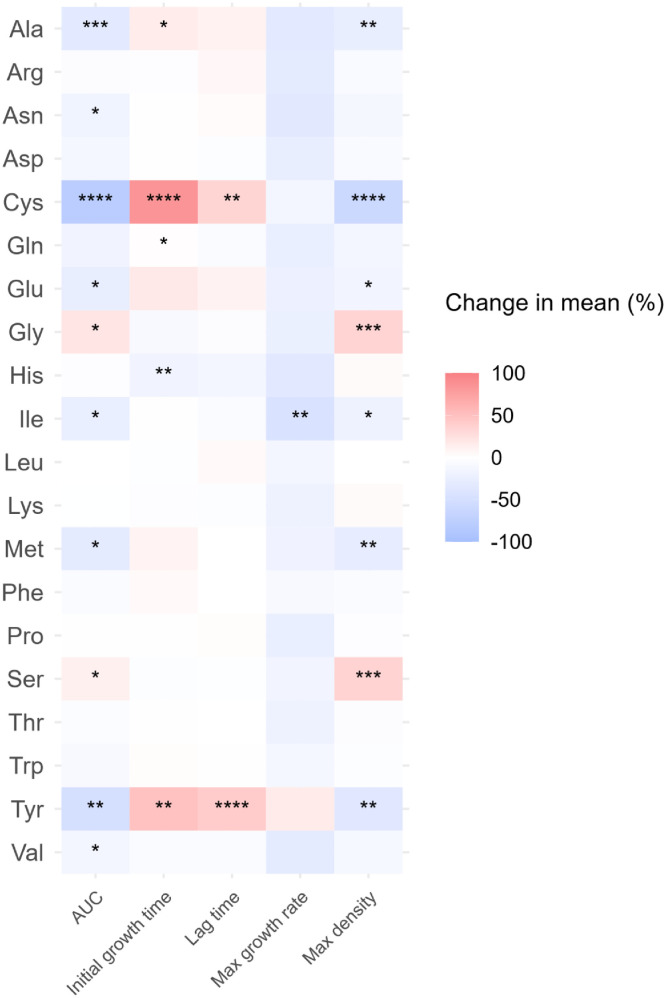
Heatmap presenting the change in mean value of five growth curves parameters (area under the curves (AUC), initial growth time, lag time, maximum growth rate and maximum density) of *S. aureus*. Ala: l-alanine, Arg: l-arginine, Asn: l-asparagine, Asp: l-aspartic acid, Cys: l-cysteine, Gln: l-glutamine, Glu: l-glutamic acid, Gly: glycine, His: l-histidine, Ile: l-isoleucine, Leu: l-leucine, Lys: l-lysine, Met: l-methionine, Phe: l-phenylalanine, Pro: l-proline, Ser: l-serine, Thr: l-threonine, Trp: l-tryptophan, Tyr: l-tyrosine, Val: l-valine. *: *p* ≤ 0.05; **: *p* ≤ 0.01; ***: *p* ≤ 0.001; ****: *p* ≤ 0.0001.

Three amino acids, being Cys, Met and Tyr, suppressed the growth of *S. aureus* (Figure 7). Total growth increased with the addition of Gly and Ser by 20.3 % (5.59 OD₆₀₀·h) and 11.8 % (3.26 OD₆₀₀·h), respectively (Figure 8). Gly and Ser also increased the mean maximum density by 35.2 % (OD_600_ of 0.345) and 34.8 % (OD_600_ of 0.350), respectively. Additionally, His promoted earlier growth, reducing the initial growth time by 16.4 % (1.50 hours).

Conversely, growth decreased with the addition of Ala (32.7 % [9.04 OD₆₀₀·h]), Asn (17.7 % [4.88 OD₆₀₀·h]), Cys (79.3 % [21.9 OD₆₀₀·h]), Glu (16.4 % [4.54 OD₆₀₀·h]), Ile (25.1 % [6.92 OD₆₀₀·h]), Met (30.4 % [8.38 OD₆₀₀·h]), Tyr (49.5 % [12.8 OD₆₀₀·h]) and Val (13.5 % [3.71 OD₆₀₀·h]). Ala increased the initial growth time by 15.2 % (1.39 hours) and decreased the maximum density by 25.8 % (0.257 OD_600_). Cys increased the initial growth time and lag time by 84.8 % (7.78 hours) and 33.4 % (3.12 hours), respectively, while reducing the maximum density by 60.5 % (0.601 OD_600_).

Gln increased the initial growth time by 1.82 % (0.167 hours), whereas Glu decreased the maximum density by 15.7 % (0.156 OD_600_). Ile reduced both the maximum growth rate and maximum density by 44.4 % (0.351) and 20.4 % (20.2 OD_600_), respectively. Met decreased the maximum density by 28.8 % (0.286 OD_600_). Tyr increased the initial growth time and lag time by 48.9 % (4.78 hours) and 40.9 % (2.64 hours), respectively, while reducing the maximum density by 36.6 % (0.350 OD_600_).

## Discussion

This study revealed the promoting or inhibiting effects of some proteinogenic amino acids on chicken pathogens in an *in vitro* model with minimal nutrient medium (MNM). Results indicated that Cys has a strong growth-inhibitory effect on *E. coli, C. perfringens, S. enterica*. and *S. aureus*; Gln and Glu promote C*. perfringens*; Gly and Ser promote *S. aureus*. In contrast, the amino acids have little effect on the three *Enterococcus* species. *(E. cecorum, E. faecalis* and *E. faecium*). Three species, which are *E. coli* (Yang et al., 2015), *S. enterica* (Ben-Porat et al., 2024) and *S. aureus* (Wyon and McLeod, 1923), had been previously studied for their response to amino acids in an *in vitro* model. However, our results do not always align with these earlier studies. The effects of amino acids on *E. coli* strain MG1655, a human-origin strain, in the M9 medium have been previously studied (Yang et al., 2015). It was shown that the growth of *E. coli* strain MG1655 was inhibited by Ser, Cys, Leu and Val (Yang et al., 2015). In our study, the APEC was inhibited by Cys and suppressed by His and Phe. Additionally, the inhibitory effects of Arg and Cys on *Salmonella enterica* strain 14028 s have been previously reported (Ben-Porat et al., 2024). Although the inhibitory effect of Cys was also observed in our study, Arg did not inhibit the growth of *S. enterica* These discrepancies can be attributed to variation between bacterial strains, where the variation of amino acid utilisation has been associated with toxigenicity, antibiotic-resistance and host origin of the bacteria (Liu et al., 2020). However, our research does not explore the response of bacteria not isolated from diseased chickens. Therefore, our results are limited to pathogenic strains from chickens and may not represent every strain within the tested species.

The amino acid concentrations tested (1 g/L) in our study were designed to explore their potential effects on bacteria in the hindgut. In one study, chickens fed with 40 g/kg of crystalline glycine exhibited concentrations of approximately 1.0, 0.6 and 1.8 g/kg in the jejunum, ileum and caecum, respectively (Dahiya et al., 2007). Initially, the experiments were conducted at a higher concentration, but Tyr solidifies at concentrations of 2 g/L and above during the incubation. Thus, as a matter of consistency, all amino acids were tested at 1 g/L. Therefore, the elevated dosage was selected to support the proof-of-concept nature of the experiment.

Among the different growth-modulating effects, the inhibitory effect of Cys is consistently reported in our study and some earlier studies (Wang et al., 2019; Ben-Porat et al., 2024). The inhibitory property of Cys was observed with a concentration-dependent manner (Kari et al., 1971). At low concentrations, Cys inhibited bacterial growth by disrupting biochemical pathways in an MNM. At concentrations below 0.2 mM, Cys was shown to disrupt the biosynthetic pathway of leucine, isoleucine, threonine and valine (Kari et al., 1971). However, these inhibitory effects are typically attributed to negative feedback inhibition of individual amino acid biosynthesis pathways and can be neutralised by other amino acids. For example, adding Thr can reverse the inhibitory effect of Cys on Ile biosynthesis in *E. coli* (Harris, 1981). In contrast, Cys may cause direct damage to the cellular structure at high concentrations. At concentrations above 2 mM, l-Cys damages the bacterial cell membrane (Kari et al., 1971; Wang et al., 2019). Our study tested Cys at 1 g/L, which is approximately 8.25 mM. This concentration may inhibit bacteria due to the destruction of the cell membrane. However, this bactericidal activity has never been reported from *in vivo* experiments. In an *in vivo* environment, even Cys (which is one of the least abundant amino acids in chicken diets) and all the other 20 proteinogenic amino acids are constantly high in concentration in the chicken intestinal tract (Wu, 2014). The interaction between amino acids in an *in vivo* environment may diminish the inhibitory effects. The practical application of Cys on living chickens needs to be further researched.

Previous *in vivo* studies correlated dietary Gly, provided via fishmeal, with growth stimulation of *C. perfringens* (Drew et al., 2004; Dahiya et al., 2007). In contrast, our study did not observe significant growth-promoting effects from Gly. Instead, *C. perfringens* was promoted by Gln and Glu, a finding not reported and divergent from earlier *in vivo* studies (Drew et al., 2004; Dahiya et al., 2007). Notably, Gln and Glu were present at twice the concentration in fishmeal compared to soybean meal whilst the concentrations (g/kg) of Gly are similar (Macelline et al., 2021). Although Glu and Gln were not reported for promoting effects on *C. perfringens* in *in vivo* studies, the amino acid profile of the fishmeal aligned with our result that Gln and Glu promote *C. perfringens* growth. The mechanisms underlying the discrepancy between *in vivo* and *in vitro* studies remain unclear and warrant further investigation.

In our study, amino acids changed the growth curves in three ways. Firstly, additional amino acids reduced the duration of the lag phase and shifted the growth curves toward zero hours of incubation in some cases. The lag phase in bacteria is a period for bacteria to adapt to a new environment (Bertrand, 2019). In other words, the closer to the optimal environment for bacteria to perform metabolism and protein transcription, the shorter the lag phase (Bertrand, 2019). In our study, the lag phase of the APCE, *C. perfringens* and *S. enterica* was shortened by Asn, Gln. Glu and Pro. Hence, it can be concluded that Asn, Gln. Glu and Pro can optimise the environmental conditions for bacterial growth. Secondly, the amino acids either increased or decreased the maximum density. A reduction in maximum density of growth curves was also reported in *Pseudomonas aeruginosa* exposed to sub-minimal inhibitory concentration (sub-MIC) levels of ceftazidime, levofloxacin and tetracycline (Sanz‐García et al., 2022). This reduction in maximum density may reflect the growth-inhibiting effect at a sub-MIC level. Similarly, in our study, Cys reduced the maximum density of *C. perfringens*, suggesting that Cys may have been present at a sub-MIC level for this species. Thirdly, the amino acids influenced the maximum growth rate. Bacterial growth rate can be affected by energy production, nutrient transport, cell wall and membrane synthesis, all of which are linked to protein abundance (Belliveau et al., 2021). The protein synthesis can be affected by the presence of amino acids. For example, protein synthesis was repressed by Ile in human-associated *S. aureus* (Kaiser et al., 2018). Specifically, Ile represses the biosynthesis of branched-chain amino acids (Ile, Leu and Val), hence reducing the bacterium’s ability of environmental adaptation (Kaiser et al., 2018). In our study, *S. aureus* also exhibited a reduced maximum growth rate in the presence of Ile, which may be a consequence of this repression.

A limitation of our study is that the effects of amino acids at different concentrations were not investigated. We tested all the amino acids at 1 g/L. This concentration may produce results that help to predict their effect in the intestine of chickens fed with encapsulated amino acids. However, it does not reveal the concentration-dependent effects. We hypothesised that there would be a therapeutic window for some amino acids. Those amino acids may promote bacterial growth below a specific concentration as a nutrient and/or inhibit at extremely high concentrations as a bactericidal. Research about the effect of amino acids at different concentrations is warranted.

This study revealed the suppressive effects of amino acids on pathogenic bacteria. The result represents the direct effects on bacterial growth. However, bacteria are mostly populated in the caecum (Shang et al., 2018). Dietary amino acids for chicken are mostly absorbed in the duodenum and do not reach the caecum (Apajalahti and Vienola, 2016). Consequently, their direct effects on caecal bacteria in the caecum are limited. To enable delivery of amino acids to the caecal microbiota, novel feeding technologies that reduce absorption in the small intestine are required. The findings of this research provide a foundation for selecting amino acids for target feed formation aimed at modulating chicken caecal microbiota.

## Conclusion

In conclusion, amino acids in the MNM can promote, suppress or have no effect on bacterial growth. Our study suggested that the inhibitory effects of Cys can be found on *E. coli, C. perfringens, S. enterica* and *S. aureus*. On the other hand, Gln and Glu promote the growth of *C. perfringens*, and Gly and Ser promote the growth of *S. aureus*. With the knowledge of growth modulation effects of amino acids on chickens’ bacteria, there might be potential application for their usage in controlling the pathogens in chickens. However, the application in controlling bacteria in a nutritional complex environment needs to be further researched.

## Funding

The study was part of the 10.13039/501100009207AgriFutures Chicken Meat Consortium – Nutrition, Gut Health and Environment project (PRO-016111) which is funded and supported by the AgriFutures Chicken Meat Program with its associated levy payers, five research providers, seven industry partners and nine international universities.

## Disclosures

The authors declare that they have no known competing financial interests or personal relationships

## Appendix Supplementary materials

Image, application 1

Image, application 2

Image, application 3

Image, application 4

Image, application 5

Image, application 6

Image, application 7

## Footnotes

Supplementary material associated with this article can be found, in the online version, at doi:10.1016/j.psj.2025.106121.

## References

[bib0001] Adedokun S.A., Olojede O.C. (2019). Optimizing gastrointestinal integrity in poultry: the role of nutrients and feed additives. Front. Vet. Sci..

[bib0002] Alazzam B., Bonnassie-Rouxin S., Dufour V., Ermel G. (2011). MCLMAN, a new minimal medium for *Campylobacter jejuni* NCTC 11168. Res. Microbiol..

[bib0003] Amos H., Cohen G.N. (1954). Amino acid utilization in bacterial growth. 2. A study of threonine-isoleucine relationships in mutants of *Escherichia coli*. Biochem. J..

[bib0004] Apajalahti J., Vienola K. (2016). Interaction between chicken intestinal microbiota and protein digestion. Anim. Feed Sci. Technol..

[bib0005] Beaumont M., Roura E., Lambert W., Turni C., Michiels J., Chalvon-Demersay T. (2022). Selective nourishing of gut microbiota with amino acids: a novel prebiotic approach?. Front. Nutr..

[bib0006] Belliveau N.M., Chure G.., Hueschen C.L., Garcia H.G., Kondev J., Fisher D.S., Theriot J.A., Phillips R. (2021). Fundamental limits on the rate of bacterial growth and their influence on proteomic composition. Cell Syst.

[bib0007] Benjamini Y., Hochberg Y. (1995). Controlling the false discovery rate: a practical and powerful approach to multiple testing. J. R. Stat. Soc. Ser. B Methodol..

[bib0008] Ben-Porat N., Ohayon A., Rosenberg T., Musa A., Petersen E., Mills E. (2024). Utilizing nutrient type compounds as anti-bacterial compounds: arginine and cysteine inhibit *Salmonella* survival in egg white. Front. Bioeng. Biotechnol..

[bib0009] Bertrand R.L. (2019). Lag phase is a dynamic, organized, adaptive, and evolvable period that prepares bacteria for cell division (W Margolin, Ed.). J. Bacteriol..

[bib0010] Blazanin M. (2023). gcplyr: an R package for microbial growth curve data analysis. http://biorxiv.org/lookup/doi/10.1101/2023.04.30.538883.

[bib0011] Dahiya J.P., Hoehler D.., Van Kessel A.G., Drew M.D. (2007). Dietary encapsulated glycine influences *Clostridium perfringens* and lactobacilli growth in the gastrointestinal tract of broiler chickens. J. Nutr..

[bib0012] De Felice M., Squires C., Levinthal M., Guardiola J., Lamberti A., Iaccarino M. (1977). Growth inhibition of *Escherichia coli* K-12 by L-valine: a consequence of a regulatory pattern. Mol. Gen. Genet. MGG.

[bib0013] Devriese L.A., Hommez J.., Wijfels R., Haesebrouck F. (1991). Composition of the enterococcal and streptococcal intestinal flora of poultry. J. Appl. Bacteriol..

[bib0014] Drew M.D., Syed N..A., Goldade B.G., Laarveld B., Van Kessel A.G. (2004). Effects of dietary protein source and level on intestinal populations of *Clostridium perfringens* in broiler chickens. Poult. Sci..

[bib0015] Duan X., Huang X., Wang X., Yan S., Guo S., Abdalla A.E., Huang C., Xie J. (2016). L-serine potentiates fluoroquinolone activity against *Escherichia coli* by enhancing endogenous reactive oxygen species production. J. Antimicrob. Chemother..

[bib0016] Foster N., Kyriazakis I., Barrow P. (2021).

[bib0017] Fuchs A.-R., Bonde G.J. (1957). The nutritional requirements of *Clostridium perfringens*. Microbiology.

[bib0018] Geldart K., Kaznessis Y.N. (2017). Characterization of class IIa bacteriocin resistance in *Enterococcus faecium*. Antimicrob. Agents Chemother..

[bib0019] Harris C.L. (1981). Cysteine and growth inhibition of *Escherichia coli*: threonine deaminase as the target enzyme. J. Bacteriol..

[bib0020] Hubert S.M., Al-Ajeeli M.., Bailey C.A., Athrey G. (2019). The role of housing environment and dietary protein source on the gut microbiota of chicken. Animals.

[bib0021] Kaiser J.C., King A..N., Grigg J.C., Sheldon J.R., Edgell D.R., Murphy M.E.P., Brinsmade S.R., Heinrichs D.E. (2018). Repression of branched-chain amino acid synthesis in *Staphylococcus aureus* is mediated by isoleucine via CodY, and by a leucine-rich attenuator peptide (DA Garsin, Ed.). PLOS Genet.

[bib0022] Kari C., Nagy Z., Kovacs P., Hernadi F. (1971). Mechanism of the growth inhibitory effect of Cysteine on *Escherichia coli*. J. Gen. Microbiol..

[bib0023] Kers J.G., Velkers F..C., Fischer E.A.J., Hermes G.D.A., Stegeman J.A., Smidt H. (2018). Host and environmental factors affecting the intestinal microbiota in chickens. Front. Microbiol..

[bib0024] Koch A.L. (1970). Turbidity measurements of bacterial cultures in some available commercial instruments. Anal. Biochem..

[bib0025] Kwoji I.D., Okpeku M.., Adeleke M.A., Aiyegoro O.A. (2022). Formulation of chemically defined Media and growth evaluation of *ligilactobacillus salivarius* ZJ614 and *limosilactobacillus reuteri* ZJ625. Front. Microbiol..

[bib0026] Liu Y.-K., Kuo H.-C., Lai C.-H., Chou C.-C. (2020). Single amino acid utilization for bacterial categorization. Sci. Rep..

[bib0027] Macelline S.P., Chrystal P..V., Liu S.Y., Selle P.H. (2021). The dynamic conversion of dietary protein and. Amino Acids into Chicken-Meat Protein. Animals.

[bib0028] Macelline S.P., Kidd M..T., Chrystal P.V., Toghyani M., Selle P.H., Liu S.Y. (2023). The influence of non-bound amino acid inclusions and starch-protein digestive dynamics on growth performance of broiler chickens offered wheat-based diets with two crude protein concentrations. Anim. Nutr..

[bib0029] Machado H., Weng L.L., Dillon N., Seif Y., Holland M., Pekar J.E., Monk J.M., Nizet V., Palsson B.O., Feist A.M. (2019). Strain-specific metabolic requirements revealed by a defined minimal medium for systems analyses of *Staphylococcus aureus* (CM Dozois, Ed.). Appl. Environ. Microbiol..

[bib0030] Milgroom M.G. (2023).

[bib0031] Mukherjee S., Bassler B.L. (2019). Bacterial quorum sensing in complex and dynamically changing environments. Nat. Rev. Microbiol..

[bib0032] Myers J.A., Curtis B..S., Curtis W.R. (2013). Improving accuracy of cell and chromophore concentration measurements using optical density. BMC Biophys.

[bib0033] R Core Team (2023). https://www.R-project.org/.

[bib0034] Riha, W. E., .and M. Solberg. 1971. Chemically defined medium for the growth of *Clostridium perfringens*. 10.1128/am.22.4.738-739.1971 PMC376401 4331776

[bib0035] Rowley D. (1953). Inhibition of E. coli strains by amino-acids. Nature.

[bib0036] Royston J.P. (1982). An extension of Shapiro and Wilk’s W test for normality to large samples. Appl. Stat..

[bib0037] Rudin L., Sjöström J.-E., Lindberg M., Philipson L. (1974). Factors affecting competence for transformation in *Staphylococcus aureus*. J. Bacteriol..

[bib0038] Sambrook J., Russell D.W. (2001).

[bib0039] Sanz-García F., Hernando-Amado S., Martínez J.L. (2022). Evolution under low antibiotic concentrations: a risk for the selection of *Pseudomonas aeruginosa* multidrug-resistant mutants in nature. Environ. Microbiol..

[bib0040] Shang Y., Kumar S., Oakley B., Kim W.K. (2018). Chicken gut microbiota: importance and detection technology. Front. Vet. Sci..

[bib0041] Souillard R., Laurentie J., Kempf I., Le Caër V., Le Bouquin S., Serror P., Allain V. (2022). Increasing incidence of *Enterococcus*-associated diseases in poultry in France over the past 15 years. Vet. Microbiol..

[bib0042] Swayne D.E. (2020).

[bib0043] Syed M.A., Ullah H.., Tabassum S., Fatima B., Woodley T.A., Ramadan H., Jackson C.R. (2020). Staphylococci in poultry intestines: a comparison between farmed and household chickens. Poult. Sci..

[bib0044] Thomrongsuwannakij T., Blackall P.J., Djordjevic S.P., Cummins M.L., Chansiripornchai N. (2020). A comparison of virulence genes, antimicrobial resistance profiles and genetic diversity of avian pathogenic *Escherichia coli* (APEC) isolates from broilers and broiler breeders in Thailand and Australia. Avian Pathol.

[bib0045] Bello U., Idrus A.Z., Yong Meng G., Awad E.A., Soleimani Farjam A. (2018). Gut microbiota and transportation stress response affected by tryptophan supplementation in broiler chickens. Ital. J. Anim. Sci..

[bib0046] Wang H., Qian J., Gu J., Yan W., Zhang J. (2019). Steric configuration-enabled selective antimicrobial activity of chiral cysteine. Biochem. Biophys. Res. Commun..

[bib0047] Wang J., Yan D., Dixon R., Wang Y.-P. (2016). Deciphering the principles of bacterial nitrogen dietary preferences: a strategy for nutrient containment (CS Harwood, Ed.). mBio.

[bib0048] Wu G. (2014). Dietary requirements of synthesizable amino acids by animals: a paradigm shift in protein nutrition. J. Anim. Sci. Biotechnol..

[bib0049] Wyon G.A., McLeod J.W. (1923). Preliminary note on inhibition of bacterial growth by amino-acids. Epidemiol. Infect..

[bib0050] Yang Y., Pollard A.M., Höfler C., Poschet G., Wirtz M., Hell R., Sourjik V. (2015). Relation between chemotaxis and consumption of amino acids in bacteria. Mol. Microbiol..

