# Supplementary material for: Use of non-bound proteinogenic amino acids to modulate the growth of pathogenic bacteria from broiler chickens
Source: Poult Sci. 2025 Nov 15;105(1):106121. doi: 10.1016/j.psj.2025.106121 (PMC12720363; doi:10.1016/j.psj.2025.106121)
Supplement: Supplementary file 5 [file mmc5.docx]

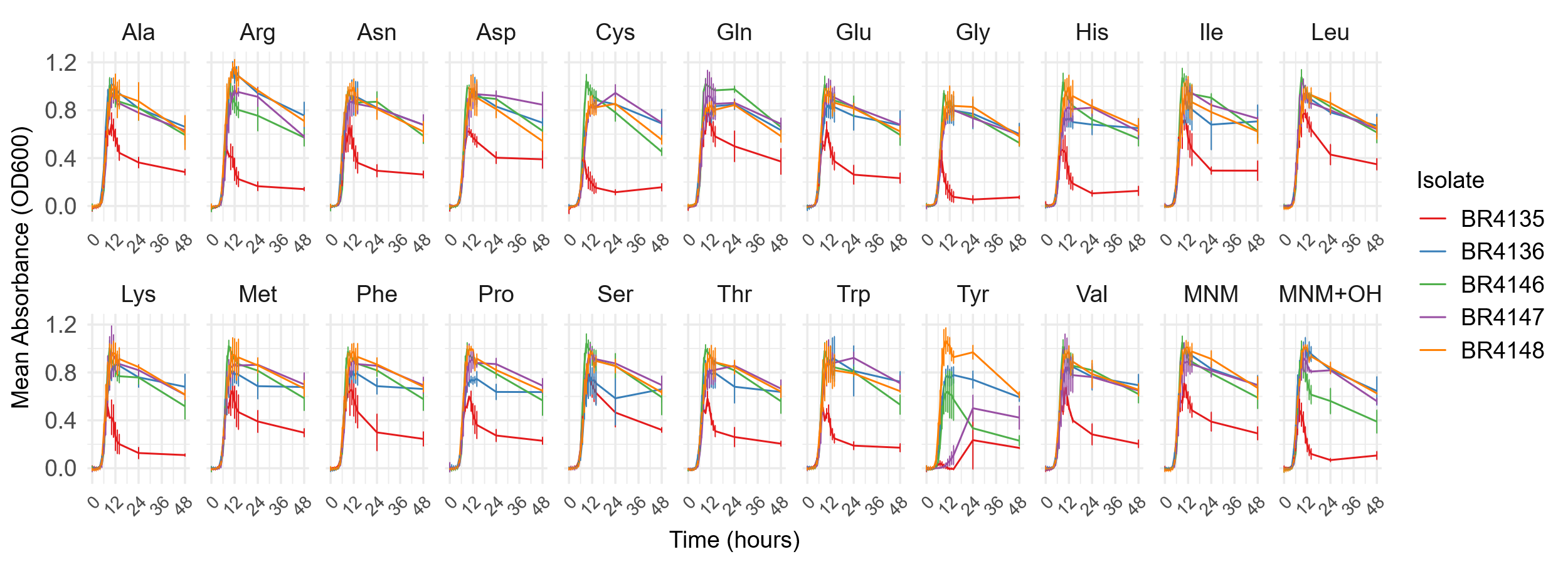


Supplementary Figure 5. Growth curves of the mean absorbance (OD_600_) of a triplicate against the incubation duration (hour) for 5 *E. faecium* isolates in MNM with or without 1g/L of each amino acid. Ala: L-alanine, Arg: L-arginine, Asn: L-asparagine, Asp: L-aspartic acid, Cys: L-cysteine, Gln: L-glutamine, Glu: L-glutamic acid, Gly: glycine, His: L-histidine, Ile: L-isoleucine, Leu: L-leucine, Lys: L-lysine, Met: L-methionine, Phe: L-Phenylalanine, Pro: L-proline, Ser: L-serine, Thr: L-threonine, Trp: L-tryptophan, Tyr: L-tyrosine, Val: L-valine, MNM (minimal nutrient medium), MNM+OH (minimal nutrient medium with 0.02*N* NaOH).
