# Supplementary material for: Use of non-bound proteinogenic amino acids to modulate the growth of pathogenic bacteria from broiler chickens
Source: Poult Sci. 2025 Nov 15;105(1):106121. doi: 10.1016/j.psj.2025.106121 (PMC12720363; doi:10.1016/j.psj.2025.106121)
Supplement: Supplementary file 6 [file mmc6.docx]

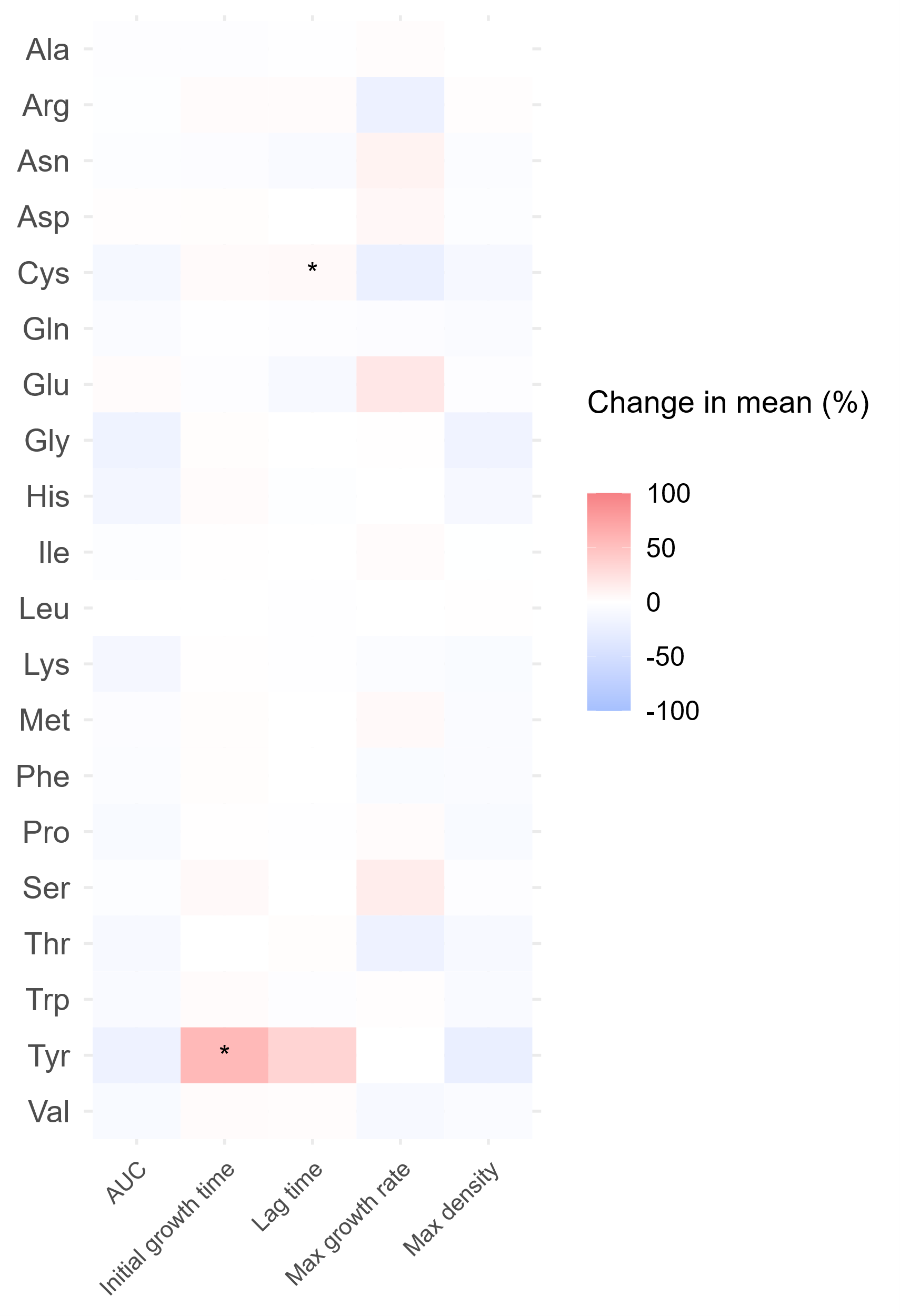


Supplementary Figure 6. Heatmap presenting the change in mean value of five growth curves parameters (area under the curves (AUC), initial growth time, lag time, maximum growth rate and maximum density) of *E. faecium*. Ala: L-alanine, Arg: L-arginine, Asn: L-asparagine, Asp: L-aspartic acid, Cys: L-cysteine, Gln: L-glutamine, Glu: L-glutamic acid, Gly: glycine, His: L-histidine, Ile: L-isoleucine, Leu: L-leucine, Lys: L-lysine, Met: L-methionine, Phe: L-Phenylalanine, Pro: L-proline, Ser: L-serine, Thr: L-threonine, Trp: L-tryptophan, Tyr: L-tyrosine, Val: L-valine. *: *p* ≤ 0.05; **: *p* ≤ 0.01; ***: *p* ≤ 0.001; ****: *p* ≤ 0.0001.
